# Telementoring for surgical training in low-resource settings: a systematic review of current systems and the emerging role of 5G, AI, and XR

**DOI:** 10.1007/s11701-025-02703-9

**Published:** 2025-08-28

**Authors:** Maurine Chepkoech, Bessie Malila, Joyce Mwangama

**Affiliations:** 1 Department of Electrical Engineering, University of Cape Town, Rhondebosch, Cape Town, 7700 South Africa; 2 Division of Biomedical Engineering, University of Cape Town, Anzio Road, Cape Town, 7925 South Africa

**Keywords:** Telementoring, Surgical training, Underserved settings, 5G, Artificial intelligence, Extended reality

## Abstract

Telementoring in surgical training enables expert surgeons to provide real-time remote guidance to trainees. This technique is increasingly adopted to address surgical training gaps in low- and middle-income countries (LMICs), i.e., nations with a gross national income per capita of $13,845 or less. However, existing systems are constrained by conventional communication systems, which experience high latency, limited bandwidth, and poor video resolution. These challenges hinder the integration of immersive and interactive technologies such as virtual reality (VR), augmented reality (AR), and haptic feedback, which would enhance learning outcomes, especially in resource-constrained environments. AR overlays digital images onto the real-world environment, whereas VR immerses users in a fully computer-generated environment, enabling trainees to interact with surgical components as if they are in the real world. In addition, the advent of the fifth generation of mobile networks (5G), which delivers ultra-low latency, high bandwidth, and support for network slicing, offers a promising foundation for scaling high-fidelity telementoring systems. Moreover, the integration of artificial intelligence (AI), i.e., equipping computer systems with the ability to perform tasks that would typically require human intelligence, can enable real-time performance analytics and skill assessment of the trainees. In regions lacking reliable network backbone infrastructure, such as fiber, hybrid approaches that combine low-cost 5G deployments with satellite communication can be leveraged to achieve reliable end-to-end connectivity. Therefore, this systematic literature review evaluates current surgical telementoring systems, their enabling technologies, and associated challenges, with emphasis on LMIC contexts, where such systems will have the greatest benefits. Through a structured Population, Intervention, Comparison, and Outcome (PICO)-based synthesis, we address five key research questions that span key aspects such as current telementoring systems and technologies, functional and technical requirements, educational outcome effectiveness, and the gaps addressed by modern technology and connection solutions, i.e., 5G, AI, VR, and AR. In addition, we identify recurrent challenges and propose a design framework that can be adopted in low-resource settings. Finally, we outline future development directions, including AI-driven evaluation models, scalable system architectures, and policy frameworks to guide the development of secure, cost-effective, and equitable telementoring platforms to advance global surgical education.

## Introduction

Worldwide, an estimated five billion people lack access to adequate surgical care, with low- and middle-income countries (LMICs) requiring an additional 143 million life-saving surgical procedures annually [1]. LMICs are nations classified by the World Bank based on gross national income per capita as having lower or moderate income levels, often facing greater resource and infrastructure constraints. According to research done in Ref. [2], approximately 94% of individuals in LMICs lack access to surgical procedures compared to 14.9% in high-income countries (HICs). HICs are nations with a high gross national income per capita, typically characterized by advanced economies, stronger infrastructure, and greater access to resources. LMICs include countries such as India, Nigeria, and Kenya, while examples of HICs include the United States, Germany, and Japan [3]. Many African hospitals are under-resourced and understaffed, and as a result would benefit from global efforts and solutions to improve surgical access. Surgical telementoring has emerged as a potential solution, providing remote access to advanced surgical expertise and enhancing the professional growth of local surgeons [4]. This concept originated from the early applications of telemedicine and telerobotics, where remote consultations and procedures were performed over long distances [5]. The first major advancements in telementoring were seen in military medicine and space exploration, where geographically isolated surgeons required real-time expertise to manage complex cases [1]. Overall, the implementation of telementoring solutions in LMICs promises to enhance access to surgical care, optimize healthcare delivery, and reduce the disease burden associated with unattended surgical conditions [6]. Through remote supervision and continuous learning, telementoring enhances surgical training and professional development, which enhances knowledge sharing and global collaboration between trainees and experts [7].

Despite the desirable contributions of telementoring to surgical access in LMICs, many challenges make existing systems suboptimal for real-time surgical training and complex procedures. These include: unreliable network connectivity, long latency, poor video resolution, image distortion [8], unreliable power supply [9], inadequate digital health policies, high equipment costs, and low healthcare expenditure [1, 5, 10–13]. However, during the past two decades, technological advancements in video streaming, artificial intelligence (AI), augmented reality (AR), and telesurgery systems have significantly improved the feasibility and effectiveness of telementoring [7, 12]. AI technology enables computers and machines to simulate human learning, comprehension, problem-solving, decision-making, and creativity, whereas AR overlays computer-generated images onto the real world, enhancing the user’s perception of reality and immersion in the virtual environments.

The earliest form of surgical telementoring was based on live video streaming and two-way audio communication, allowing mentors to instruct mentees verbally during training procedures. However, high latency, poor image resolution, and limited internet bandwidth in remote areas often compromise its effectiveness [13]. Second in the evolution was telestration [12]. This technique enabled mentors to annotate surgical images and live video feeds in real time, providing clearer guidance on procedural steps and instrument handling for the mentees. Thirdly, the teleassistance technique was developed as an advanced form of telestration. Teleassistance integrates robotic and haptic feedback systems, allowing mentors to remotely guide the hands of trainees using robotic arms or augmented haptic interfaces. This enables real-time force feedback, whereby the mentees can feel the consistency of tissues, improving their precision and psychomotor skills. Currently, the latest developments in telementoring systems entail improving the trainee’s perception of reality through the integration of extended reality (XR) capabilities [14]. XR is a collective term for immersive technologies that include: virtual reality (VR), AR, and mixed reality (MR) to create immersive and interactive learning environments. VR places users in a fully computer-generated environment, whereas AR overlays digital information onto the real world, and MR merges the physical and virtual worlds so that digital and real objects can interact in real time. XR-enhanced systems overlay digital instructions on anatomical image casts, which enhances spatial awareness, allowing the mentees to practice complex procedures in low-risk, simulated environments [13]. For instance, in Ref. [12], an example of an XR-enhanced telementoring solution, i.e., the RMS-XR system, is presented. The system integrates 2D endoscopic streaming, 3D reconstructed surgical scenes, and stereo occlusion-aware overlaid guidance within an immersive XR environment. In its evaluation, the system achieved high user-reported immersion and confidence, and demonstrated practical feasibility for robotic surgery with setup times under 5 min. Current developments in telementoring systems include proposals for AI-driven and edge-computing-based systems that leverage AI-powered skill assessment and real-time analytics to enhance training quality. In this context, AI in surgical education and training has been defined as the use of an intelligent program that acts to fulfill or support the fulfillment of educational tasks traditionally performed exclusively by surgical educators, through making decisions like educators, and providing customized adaptation, including performance assessment and feedback to surgical trainees [15].

Telementoring in HICs has evolved into a highly sophisticated and well-integrated component of surgical education and practice. Many leading medical institutions, such as Johns Hopkins University (USA), Mayo Clinic (USA), Imperial College London (UK), and University of Toronto (Canada), have successfully implemented surgical telementoring programs. For instance, Shin et al. in Ref. [16] developed a novel robotic-assisted telementoring interface, demonstrating its impact on surgical training at Johns Hopkins University, while Hinata et al. in Ref. [17] investigated a telementoring system for robot-assisted radical prostatectomy, showing significant improvements in the learning curve. The success of these systems in HICs comes from the presence of high-speed broadband, fiber optic networks, and 5G infrastructure that enable immersive, interactive, real-time, high-resolution video streaming, telestration, and robotic-assisted surgical mentoring. Furthermore, institutions in HICs benefit from established regulatory frameworks and policies, and stable power grids that support seamless implementation and scalability. However, these successful implementations of surgical telementoring in HICs face challenges, such as interoperability between proprietary systems, cybersecurity risks, and the high cost of system acquisition and operation.

In LMICs, simulation-based training is more prevalent [18]. Such training provides a structured and controlled environment for the development of skills while prioritizing patient safety and helping mentees improve decision-making skills. With such systems, trainees can practice complex procedures in a risk-free environment, facilitating the development of essential surgical competencies. In addition, cost-effective and portable simulation models tailored to LMICs have expanded training accessibility, empowering local healthcare providers to refine their surgical skills and improve patient outcomes. Surgical simulation has proven to be particularly valuable in training for rare and complex procedures, such as nerve-sparing retroperitoneal lymph node dissection (RPLND), where trainee exposure is typically limited [19]. A study evaluating a video-based and cadaver-simulation training model for RPLND demonstrated significant improvements in surgical performance, including higher lymph node resection rates and improved trainee technical proficiency, as assessed by expert reviewers. Similarly, the complexity of laparoscopic surgeries presents a steep learning curve for trainee surgeons, necessitating continuous and hands-on practice of tool handling and coordination [20]. Such surgeries are particularly preferred because, unlike open surgeries, smaller incisions are made and the patient experiences less pain, faster recovery, minimal scarring, and a lower risk of infections and blood loss. However, despite the advantages of laparoscopic surgeries, the learning curve is steep, resulting in a limited number of surgeons equipped with such skills.

The successful implementation of reliable telementoring systems requires robust communication systems to ensure that mentees and mentors have a real-time exchange of instructions and a view of the surgical field. However, in LMICs, existing telementoring systems rely on broadband internet, satellite communications, and, at best, the fourth generation of mobile networks (4G), which struggle to meet the high demands of real-time, high-definition video streaming and low-latency data transmissions. Unstable network connections and high latency lead to delayed feedback, reduced image resolution, and potential disruptions during critical surgical procedures and instructions [13]. Although standalone satellite connections are prevalent in rural parts of LMICs, they are often costly, suffer from high latency, and are susceptible to weather-related disruptions. Similarly, 4G networks, though widely available, face speed and reliability limitations to support high-fidelity surgical video transmissions in real time [5]. Furthermore, when incorporating AR, VR, and AI-based surgical training guidance, high-bandwidth, low-latency connections are vital.

The advent of fifth-generation mobile networks (5G) technologies presents a transformative solution to address the limitations mentioned above, as it enables more stable data transmission, up to 100 times faster than 4G, reduces latency to $<10$ms, supports network slicing, and allows multiple simultaneous device connectivity [21]. These attributes make 5G networks ideal to support advanced technologies such as AR, VR, AI, and haptic-enabled surgical training. However, the incorporation of 5G and AI into existing surgical training modalities is still a new endeavor that is being explored in modern implementations, research, and evaluations.

Therefore, the main contributions of this systematic review include the following.

- We provide a comprehensive and technically grounded resource on the state-of-the-art telementoring systems for surgical training in LMICs
- We consolidate findings on the design, implementation, and integration of 5G-enabled telementoring solutions, explore the role of XR, haptics, and AI-driven feedback systems, and highlight the challenges of network infrastructure limitations, cybersecurity risks, and regulatory barriers
- We present an ideal telementoring system design and the technical requirements to achieve collaborative, real-time, immersive, and interactive exchange of instructions between mentors and mentees
- We focus on network infrastructure, which is among the key limitations in the real-world deployment of telementoring systems, and explore the contributions of hybrid network architectures, including 5G satellite integration for reliable remote surgical mentorship in low-resource environments
- We present an interdisciplinary review that uniquely bridges surgical education, network engineering, and digital health innovations in LMICs by evaluating both the effectiveness of telementoring platforms and the technical feasibility of real-time telesurgical interventions over heterogeneous networks

**Fig. 1:**
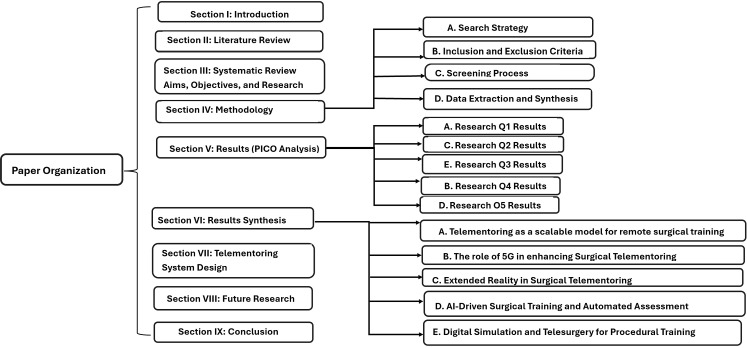
Paper organization

The organization of this systematic literature review paper is shown in Fig. 1.

## Literature review and related works

### The state of surgical expertise in LMICs

In the systematic review done in Ref. [22], the average physician-patient ratio in LMICs is approximately ten times lower than in HICs, resulting in delayed and expensive surgical care for patients in underserved regions. To address this challenge, LMICs should invest in intraoperative telemedicine solutions and improve access to specialist surgical care. Furthermore, the shortage of surgeons in LMICs highlights the need for innovative and cost-effective training methods to expand the surgical workforce more rapidly.

Currently, the conventional surgical training method in LMICs is often based on apprenticeship models, where trainees learn through direct observation and hands-on practice under the supervision of experienced surgeons [22]. In Ref. [23], the cognitive apprenticeship framework was adopted in Mozambique as a viable method to equip surgeons in LMICs with the necessary procedural skills. This method involved a mix of international collaborations and digital learning tools. However, the scarcity of qualified mentors and limited access to advanced surgical procedures can restrict the breadth, frequency, and depth of training [24]. For effective telementoring, integrating online education reduces logistical costs over wide geographical distances. Similarly, telesimulation adds value by allowing trainees to spend more time practicing simulated surgical procedures while receiving real-time feedback on their performance [25].

During the COVID-19 pandemic, when travel restrictions were enforced, training programs had to switch to remote and online methods, such as webinars [4]. However, the degree of surgical training that could be offered in this manner was mainly theoretical and observational for the trainees. As a result, hands-on skills, which are a crucial aspect of surgical expertise, were missing. The review in Ref. [4] evaluates the impact of the COVID-19 pandemic on medical education and proposes strategies to strengthen medical training in the post-pandemic era. The authors recommend hybrid learning approaches for surgeons, such as a blend of e-learning tools, XR technologies, and curriculum restructuring to build an effective healthcare workforce. XR technology comprises of immersive techniques that blend the physical and digital worlds, enabling interactive experiences by either fully immersing users in a virtual environment or overlaying digital elements onto the real world.

Digital surgical simulations have also been adopted as a tool for medical education in LMICs [18]. These simulations offer interactive platforms for trainees to practice surgical procedures virtually, improving their anatomical and procedural knowledge. A scoping review presented in Ref. [8] examined the implementation of these simulations, revealing that participants, primarily medical students and residents, rated the simulators highly in terms of acceptability and perceived usefulness to improve their practical anatomical skills. However, the study identified several challenges that hinder effective implementation, especially in resource-constrained settings. These included technical issues such as image distortion, excessive light exposure, and video stream latency, which could impede the learning experience. Furthermore, the cost of implementation was up to $6,990, which poses potential financial barriers to widespread adoption. The review also highlighted the lack of long-term monitoring and evaluation of these digital tools, raising concerns about their sustainability and impact on surgical education. In addition, many of these innovations have been proposed in HICs, suggesting a potential disconnect between the development of these tools, the practical needs, and the infrastructural contexts of LMICs.

### Scope of telehealth adoption in LMICs

Telehealth encompasses telementoring, teleconsultation, remote diagnosis, and digital health platforms, in which healthcare providers in resource-limited settings can gain access to specialized expertise without the need for physical co-location [26]. Improvements in mobile connectivity, digital platforms, and telecommunication infrastructure have significantly reduced barriers to remote mentorship, with various governments and non-governmental organizations promoting policies to integrate telemedicine into healthcare systems [8, 22].

Several telementoring models have been successfully implemented in LMICs, demonstrating improvements in surgical training, procedural precision, and access to specialized care. Such models include Project Extension for Community Healthcare Outcomes (ECHO), which has been widely used for cervical cancer prevention and treatment training through live video conferencing and case-based discussions, allowing clinicians to expand their expertise remotely [27]. Project ECHO initiative adopts a hub-and-spoke model, in which expert surgeons remotely support local providers in LMICs through interactive case-based learning. It offers a scalable and cost-effective solution to surgical training where real-time telesurgery or AR-based systems are not feasible. Similarly, the SurgTime platform has been instrumental in arthroscopy training through telesurgery and AR annotations, allowing trainees to receive real-time feedback on surgical techniques and instrument handling [28]. The application of virtual platforms for laparoscopic training has also shown promise, with studies highlighting increased laparoscopic proficiency among trainees while reducing the financial burden of in-person training programs [29, 30].

Despite the promising success of the adoption of telehealth in LMICs, there are still significant challenges to its full adoption. For example, many healthcare institutions struggle with the financial constraints of investing in high-fidelity telemedicine equipment and the necessary 5G infrastructure. There is a need for cost-effective XR-based telementoring platforms that provide high-fidelity surgical simulations at a fraction of the cost of traditional training models [8]. In addition, regulatory and ethical concerns, such as cross-border licensing issues, patient data security, and inconsistent telehealth policies, have slowed the integration of telemedicine into surgical education programs [31]. Furthermore, some healthcare professionals remain skeptical about the efficacy of remote mentoring, highlighting the need for longitudinal studies that evaluate the long-term clinical impact of telementoring and telesurgery on patient outcomes [32].

### Technological barriers and challenges of surgical training in LMICs

Infrastructure limitations, ranging from equipment, networking challenges, and electrical power constraints, hinder the effectiveness of telementoring systems in LMICs. Although HICs have embraced robotic surgery as a standard for minimally invasive procedures, the high costs of robotic consoles, maintenance, and operational training make these systems inaccessible for most LMIC hospitals [6, 33].

The systematic review presented in Ref. [22] provides a summary of real-time telementoring, telesurgical consultation, and telesurgery in surgical procedures in patients in LMICs for intraoperative telemedicine. The paper emphasizes the importance of developing standardized protocols to technologically support telementoring applications. They categorize the challenges to effective implementation of surgical telementoring systems into technological barriers, training and acceptance, sustainability, and the need for standardization of training protocols. In particular, on the technological aspect, the limited access to reliable Internet connections and advanced medical equipment in LMICs hinders the widespread adoption of telemedicine and needs further development.

Furthermore, in Ref. [25], a training simulation kit aimed at training 109 graduate anesthesiologists on surgical procedures is presented. Among the key requirements for high-fidelity interaction between mentees and mentors, there was a need to ensure real-time feedback using the simulation tool for trainees. However, the unavailability of robust communication infrastructure challenges the realization of high-fidelity models at the training locations, and the commercial ones are expensive. The authors also highlight the importance of an immersive and interactive system through the incorporation of AR, VR, AI, and haptic feedback. However, the integration of these technologies needs to be done on a need basis and also with the cost factor in mind.

Table 1 summarizes key characteristics, technologies, and infrastructural limitations of telementoring systems identified in recent reviews.

**Table 1:** Summary of selected studies on telementoring systems for surgical training in resource-constrained settings

Ref	SLR	Study details	Networking challenges	Technologies used	Other technology
[34]	Yes	Meta-analysis comparing XR training to conventional robot-assisted surgery training	Infrastructure gaps and access to XR simulators	XR simulators (VR/MR), GEARS scoring	Dry-lab simulators
[2]	No	Mentored 26 robotic prostate and renal surgeries using Proximie	Limited reliable internet in LMICs; latency concerns	Cloud video platform	In-person proctoring
[13]	Yes	Reviewed STAR, ARTEMIS, InTouch, AI-MEDIC, and TMUSMI systems	Connectivity instability and high cognitive load from suboptimal visual and audio integration	AR, VR, telestration, haptics (cybergloves, exosuits), AI-enhanced mentorship (AI-MEDIC)	Satellite, ISDN lines, 3G/4G mobile internet
[35]	No	Developed a holographic telementoring system projecting live open surgery overlay for mentor guidance	High bandwidth demand, 3D rendering latency	Hologram overlays, live video	2D video streaming
[36]	No	AR mentoring with 3D scene streaming and hand interaction validated on stereo surgical videos	Latency-sensitive loop, emphasizes bandwidth-efficient 3D streaming, WAN-agnostic	AR HMD, 3D reconstruction and streaming, hand-gesture capture, stereo overlay on console	2D video guidance and teleconferencing
[12]	No	XR remote-mentoring prototype: mentor uses OST-HMD to view live 3D endoscopic scene and provide hand or virtual-tool guidance	Low-latency, synchronized bidirectional streaming of stereo or 3D and overlay, WAN type not fixed	OST-HMD, 3D endoscopic streaming, console 3D overlay, hand-gesture input	2D video and telestration

### Contributions and novelty of this review

Despite the growing body of literature on telementoring, telesurgery, and XR for surgical education, current reviews fall short in three key dimensions critical to global surgical equity:

1. Systematic focus on low-resource settings
2. Technological integration of next-generation networking, including 5G and edge computing, and immersive technologies such as XR and haptic feedback
3. Interdisciplinary synthesis of telementoring modalities, e.g., AR, VR, MR, haptics, within real-time procedural guidance frameworks

The unique contributions of this review include:

- Explicitly targeting low-resource and underserved environments, where surgical telementoring can have the greatest impact
- Integrating the emerging role of 5G, mobile broadband, and edge connectivity to address persistent barriers such as latency, bandwidth, and network availability and reliability
- Mapping the convergence of AR, VR, and haptics with surgical mentoring workflows, an aspect that is less considered in current literature
- Highlighting the feasibility, challenges, and roadmap for adopting and scaling these technologies across diverse geographies through a systematic evidence-based analysis

## Systematic review aims, objectives, and research questions

This systematic literature review aims to analyze and synthesize existing evidence on telementoring systems for surgical training, with an emphasis on their implementation and effectiveness in resource-constrained settings. The review seeks to identify technical and infrastructural aspects that support or hinder remote surgical mentorship and assess the readiness of current systems for integration with next-generation technologies such as 5G, XR, and AI.

The following research questions guide this review:

1. What telementoring systems and platforms are currently used for surgical training, particularly in rural and LMIC contexts?
2. What are the defining characteristics and operational requirements of effective telementoring systems in surgical education?
3. How do these systems impact the acquisition of surgical skills, anatomical precision, and procedural competence?
4. What outcomes are used to assess the effectiveness of telementoring in surgical training?
5. Where do current systems fall short, and how might modern communication technologies and immersive modalities address these limitations?

These research questions were further subdivided as described in Table 2 together with their objective and scope.

**Table 2:** Systematic review research questions, objectives, and scope

Research question	Objective	Scope
Q1. What telementoring systems and platforms are currently used for surgical training, particularly in rural and LMIC contexts?	Identifies the existing telementoring tools and their success and limitations	Covers the techniques used in telementoring, specific technologies, and training methods
Q2. What are the defining characteristics and operational requirements of effective telementoring systems in surgical education?	Explores the components of a telementoring system, the technologies, and infrastructure requirements	Covers the mentor, the network, and the mentee domains of the system
Q3. How do these systems impact the acquisition of surgical skills, anatomical precision, and procedural competence?	Evaluates the trainer surgeons and the mentees’ reports, about their training experience	Examines the success and challenges of in-person training versus the telementoring cases
Q4. What outcomes are used to assess the effectiveness of telementoring in surgical training?	Explores the metrics of evaluation, e.g., improvements in tool handling, confidence, or new skill acquisition	Survey reports from studies done in evaluating the usability and success of these systems
Q5. Where do current systems fall short, and how might modern communication technologies and immersive modalities address these limitations?	It investigates the technological and infrastructural limitations of current systems	Covers the contributions of 5G, AR, haptics, AI, and XR

## Systematic review methodology

This systematic review was conducted according to the Preferred Reporting Items for Systematic Reviews and Meta-Analyses (PRISMA) guidelines, employing a structured and reproducible approach to identify and synthesize relevant literature on telementoring for surgical training in low-resource settings. The PRISMA method provides a transparent way of documenting how studies are identified, screened, deemed eligible, and included in a systematic review. Given the interdisciplinary nature of the topic, which spans surgical education, network engineering, and digital health, the search methodology was designed to capture a wide range of relevant studies across these fields.

### Search strategy

A comprehensive search strategy was developed, guided by predefined research questions and inclusion criteria. The literature was retrieved from six major databases: **PubMed, IEEE Xplore, Scopus, Web of Science, ACM Digital Library, and ProQuest**. In addition, a general Google search using the search string was conducted.

The search included peer-reviewed journal articles, conference proceedings, book chapters, and technical reports. We used the following core phrases.

- “Telementoring in surgical education”,
- “Surgical training in low-resource settings”,
- “Telesurgery and rural healthcare”,
- “Effectiveness of telementoring in surgery”,
- “Telehealth technologies for surgical training”,

The core phrases were combined using Boolean operators to give the following search string that was used on all databases: ((“telesurger*” OR “telementor*” OR haptic*) AND (“developing countr*” OR LMIC* OR “low-resource setting*”) AND (education OR training OR learning)). The search results were filtered for relevance based on the review of the title, abstract, and full text, focusing on publications made in English. The selection and analysis process is further detailed in the sub-sections that follow.

### Inclusion and exclusion criteria

We defined our inclusion and exclusion criteria based on three broad considerations, i.e., the study design, the participants and the setting of the study, and the contextual focus.

#### Inclusion criteria

**1. Study design:** Studies with the following characteristics were included:

- Peer-reviewed articles including primary research studies, systematic reviews, meta-analyses
- Studies published over the past 10 years, from 2024
- Studies that discuss telementoring systems for surgical training and mentorship
- Studies focused on low-resource settings, including rural areas and LMICs
- Studies that evaluate the effectiveness of telementoring in improving surgical skills, e.g., technical competence, procedural outcomes, and knowledge transfer
- Studies exploring the technologies that support telementoring, such as video conferencing, mobile networks, mobile applications, AR/VR/MR, or robotic systems
- Studies published in English
- Studies that discuss barriers, challenges, effectiveness, or outcomes of telementoring surgical education

**2. Participants and study setting:** We included studies involving surgical trainees, residents, or practitioners from underserved areas and research from hospitals, medical schools, or health facilities in low-resource settings.

**3. Contextual focus:** In evaluating the effectiveness and contributions of telementoring in surgical education, we included studies discussing infrastructure and technology aspects that support digital and remote surgical training.

#### Exclusion criteria

**1. Study design:** We excluded studies with the following characteristics:

- Studies not focused on surgical training or telementoring, such as studies on telemedicine for general medical care
- Studies outside LMICs or rural settings, unless they provide a unique technological insight
- Papers that are not peer-reviewed, e.g., opinion articles, editorials, etc.
- Studies that do not assess surgical skill outcomes, e.g., only focusing on theoretical knowledge
- Studies that do not discuss in detail the infrastructure and technologies used in telementoring
- Studies that are not in English

**2. Participants and Setting:** We excluded studies on nonsurgical specialties. Research on telementoring systems in HICs or those not focusing on underserved and LMIC populations was excluded.

**3. Quality and scope:** Literature with low methodological rigor, e.g., studies without clear design, low sample sizes, no comparison groups, and studies that do not provide relevant data, e.g., no measurable outcomes related to telementoring in surgery, were excluded.

### Screening process

To manage the large volume of records retrieved during the initial database search, a two-step screening process was implemented to evaluate the studies against the predefined inclusion criteria. In the first stage, *titles* and *abstracts* were screened, and in the second stage, the *full texts* of the remaining studies were reviewed to verify that they met the inclusion criteria. During this stage, studies were synthesized to assess their relevance, methodological quality, and contribution in answering the outlined research questions. In cases of ambiguity and uncertainty regarding study eligibility for inclusion, a second independent reviewer evaluated the article to reach a consensus.

**Fig. 2:**
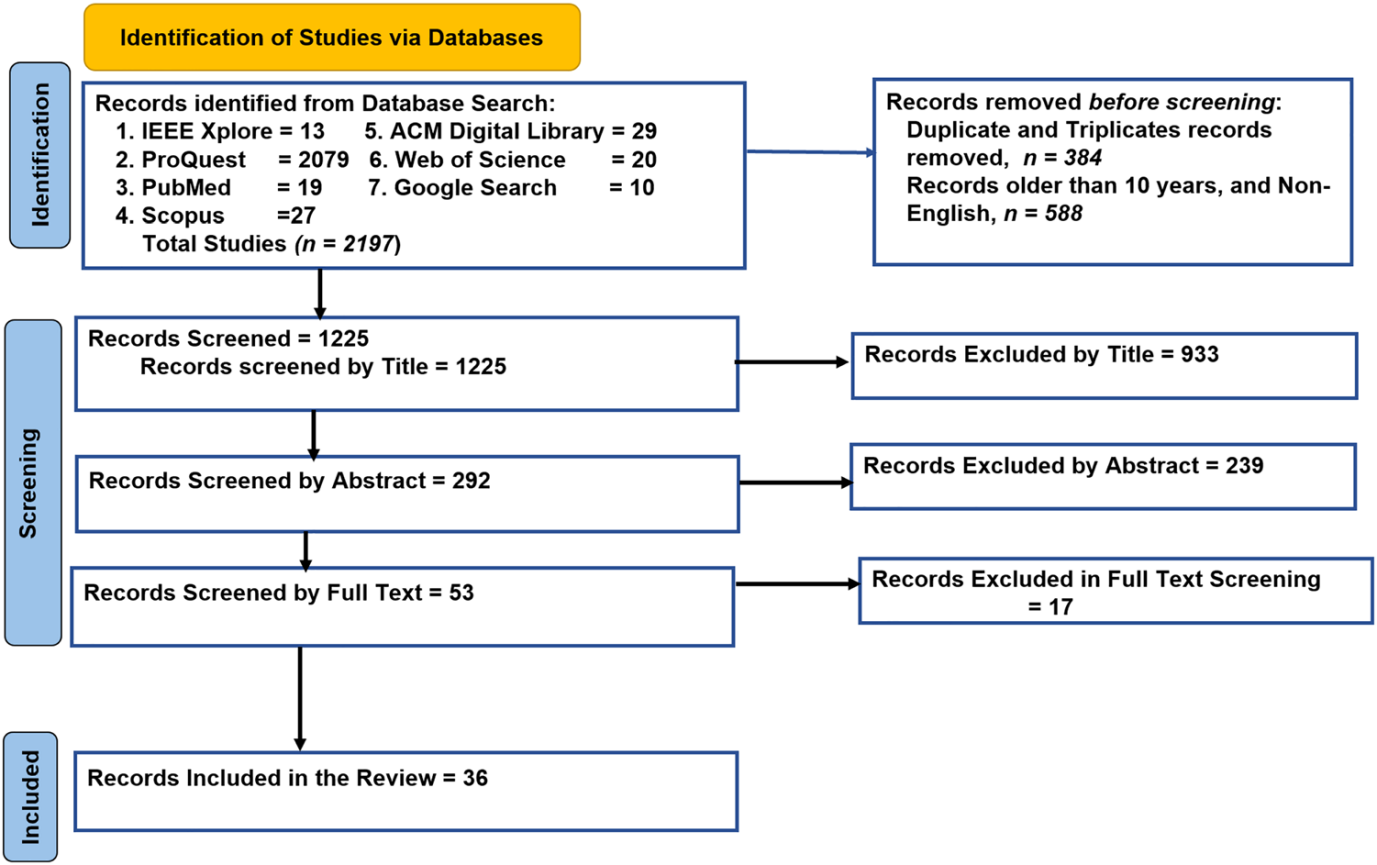
PRISMA screening diagram

Figure 2 shows the PRISMA diagram that was used to document the process for study inclusion. Initially, *2197* records were retrieved through database searches. After removal of duplicates, studies not in English, and those studies older than the past 10 years, *1225* unique records remained for screening. In the first stage, titles were reviewed, leading to the exclusion of *933* studies that were not relevant to telementoring, surgical training, or low-resource settings. The remaining *292* studies underwent abstract review, during which *239* articles were excluded. The remaining *53* studies underwent full-text review, and *17* were excluded. Ultimately, ***36*** studies were included in the final systematic review.

### Data extraction and synthesis

Data from the 36 included studies were extracted and synthesized. In the first round, an Excel sheet extraction form was used to record the details of each paper. The themes used in the inclusion and exclusion criteria, i.e., the study design, system details, the participants and setting of the study, contextual focus, outcomes, and the challenges encountered, were considered. Second, data from the included studies were extracted in the sample format outlined in Table 3. Key fields considered in the studies included were the *title, year of publication, authors, and the key theme addressed*. We then summarized the key findings and results of the studies alongside challenges and gaps identified. In addition, we took notes of the author’s conclusion and included our comments on how the research is relevant to our review. Finally, we identified and documented the potential areas of improvement or areas that need further research and development.

**Table 3:** Sample data extraction form

Title	5G-assisted telementored surgery
Author Year	Lacy et al., 2019
Full Reference	Lacy, A. M., Bravo, R., Otero-Piñeiro, A. M., Pena, R., De Lacy, F. B., Menchaca, R. & Balibrea, J. M. (2019). 5G-assisted telementored surgery. *British Journal of Surgery*, 106(12), 1576–1579. https://doi.org/10.1002/bjs.11364
Is the study eligible for inclusion? (Yes/No)	Yes
Key Theme	5G-enabled telementoring for surgery
Summary of Work	The authors demonstrated the feasibility of performing surgical telementoring using 5G networks. They used real-time audiovisual communication with telestration capabilities between operating surgeons and a remote mentor during two laparoscopic surgeries. The system leveraged high-speed 5G connectivity to minimize latency and maintain high-quality video transmission
Results	Two laparoscopic rectal surgeries were completed with real-time mentoring over a 5G network. The transmission speeds were consistently high (95–106 MB/s), latency was low (146–202 ms), and there was no significant signal loss or pixelation. Surgeons rated image quality highly ($\sim$ 9.5–9.67/10) and communication responsiveness at 10/10. No intraoperative complications occurred. The high-resolution imaging and bidirectional telestration tools allowed accurate intraoperative guidance, improving surgical precision. The trainees showed faster skill acquisition and improved tool handling compared to traditional video-based mentoring systems. However, limitations included network reliability, dependency on stable 5G connectivity, and the lack of remote haptic feedback integration
Author’s Conclusion	5G-assisted telesurgery can revolutionize remote surgical training by providing real-time, high-fidelity telementoring. With the reliable connectivity, 5G can improve the effective of real-time telementoring increasing training satisfaction among surgical teams
Relevance to This Review	The study is highly relevant as it supports the integration of 5G into surgical telementoring systems, especially for expanding access to expert mentoring in regions with limited surgical expertise. It provides empirical evidence of 5G’s capabilities in reducing latency and improving remote surgical training outcomes. It also identifies remaining barriers, such as technical, legal, and cost, that are crucial for developing sustainable telementoring programs in underserved regions
Future Research	Future research should explore the development of scalable, cost-effective telementoring frameworks tailored for LMICs. In addition, improving the network coverage in rural areas for instance using satellite communication technologies will improve adoption of telementoring in remote and underserved regions. Advancements in AI-assisted telesurgical robotics should also be explored to further augment remote surgical training capabilities. Moreover, future studies should incorporate larger and more diverse surgeon cohorts, employ standardized assessment instruments, and systematically evaluate cost-effectiveness, technical performance, and compliance with legal and ethical standards

### Application of the PICO framework

We adopted the PICO framework to systematically evaluate and synthesize the included literature. This framework is commonly used in evidence-based research to guide inclusion criteria, data extraction, and analysis of results. Through this approach, we categorized and compared studies based on four key components: Population, Intervention, Comparator, and Outcome (PICO).

**Population (P):** The population includes surgical trainees, resident doctors, medical students, and junior surgeons from low-resource settings. In some studies, the population also includes expert surgeons and patients.

**Intervention (I):** The interventions under investigation are various telementoring systems and associated technologies for remote surgical training. These include digital communication platforms, AR, VR, MR, 5G connectivity, robotic consoles, haptic devices, and AI-driven performance analytics nodes. Each study was analyzed to determine which technological components were used and how they were integrated into the telementoring systems.

**Comparator (C):** The comparator in the reviewed studies is the traditional approach to surgical training; either in-person mentorship or standard telemedicine systems, e.g., those relying on conventional broadband networks and video conferencing systems. This comparison helps to assess whether the integration of advanced technologies such as 5G, AR/VR, and AI offers measurable improvements in telesurgical training outcomes.

**Outcome (O):** Outcomes were evaluated based on several performance metrics. These include:

- Skill acquisition, including improvements in surgical dexterity and technical competence
- Procedural accuracy, such as reduction in procedural errors and enhanced precision during operations
- Training efficiency, leading to better retention of surgical skills
- User satisfaction reported by both trainees and mentors
- Where available, improved patient outcomes

## Results

We applied the PICO framework to structure the analysis of the included studies. This enabled a consistent evaluation of the focus, methodology, and findings of each study. Furthermore, this mapping informed the narrative synthesis, in which each research question is discussed.

### Q1. What telementoring systems and platforms are currently used for surgical training, particularly in LMICs?

**P:** Surgical trainees and junior surgeons operating in LMICs

**I:** Implementation of telementoring platforms, including but not limited to SurgTime, Project ECHO, Microsoft HoloLens–based AR telementoring

**C:** Conventional in-person training and mentorship models or telementoring systems in HICs

**O:** Improvement in surgical skill acquisition, better patient outcomes, and accessibility to expert mentorship

Table 4 summarizes PICO elements addressing research Q1.

**Table 4:** PICO analysis to address research Q1

Ref.	P	I	C	O
[21]	Trainees in rural and urban China	5G-enabled telementored laparoscopic surgery	Conventional in-person mentoring or no mentoring	Skill improvement, reduced complications
[37]	Surgeons and trainees in Tanzania	Video-based telementoring with on-site mentorship	Traditional non-mentored theoretical training	Increased procedural volume and improved surgical outcomes
[38]	Doctors in Texas, Latin America, Africa	Telementoring on cervical cancer screening and treatment via ECHO model	Lack of structured mentoring	Higher screening rates and treatment management
[39]	Junior physicians in rural Peru	One-on-one telementoring through video conference tools	Pre-intervention skills level	Higher procedural competence and improved confidence
[40]	Surgical trainees and practicing surgeons in India	Case-based tele-education modules, with telemedicine aspects supported	No structured remote training before intervention	Skill improvement, knowledge exchange, and improved trainee satisfaction
[28]	Orthopedic surgeons	SurgTime platform which comprises AR and classic telementoring system	Traditional hands-on mentorship	Faster skill acquisition and improved learning curves

### Q2. What are the defining characteristics and operational requirements of effective telementoring systems in surgical education?

Table 5 outlines the PICO analysis for research Q2, which includes critical system features such as ultra-low latency 5G communication, high-definition video streaming, bidirectional audiovisual feedback, AR overlays, and scalable telesimulation platforms.

**P:** Surgical trainees, surgical educators, and remote surgical mentors

**I:** Advanced telementoring system features, such as high-definition bidirectional video streaming, telestration capabilities, XR-based environments, and AI-driven procedural feedback tools, supported by low-latency and reliable network infrastructure

**C:** Traditional surgical residency and in-person training programs

**O:** Improved knowledge transfer, reduction in procedural errors, improved learner participation, and increased accessibility to specialized surgical education

**Table 5:** PICO analysis to address research Q2

Ref	P	I	C	O
[21]	Surgical trainees undergoing remote laparoscopic training	5G-enabled ultra-low latency communication with HD video (Latency $<100$ ms, 1080p video)	Traditional 4G or Wi-Fi networks	Improved image quality, reduced latency, better procedural performance
[41]	Junior surgeons in LMICs	Microsoft HoloLens-based AR platform with telestration, hands-free control over 30 fps transmission	No AR assistance	Enhanced procedural navigation, improved mentor-trainee communication
[42]	Healthcare providers in low-resource environments	Cross-platform AR system for remote procedural teaching with a low-cost design with target bandwidth $<50$ Mbps	Standard video-based training	High user satisfaction, effective knowledge transfer
[29]	Surgical trainees in LMICs	Cloud-based telesimulation platform (GLAP telesim and online modules), with bandwidth requirement range of 5–10 Mbps	Traditional simulation-only training	Scalability, accessibility improvements in training
[28]	Orthopedic trainees learning arthroscopy	AR-guided live telementoring platform, i.e., SurgTime system with frame rate of $>24$ fps minimum	Traditional in-person coaching	Higher efficiency, improved skills with remote feedback
[43]	Robotic surgical trainees	Digital twin teleoperation system for remote task simulation with a round-trip delay $<150$ ms	Conventional local simulation	High system responsiveness offering realistic training experience
[13]	Surgeons and emergency physicians	Real-time remote procedural mentoring using mixed communication technologies with a target latency of $<200$ ms	No remote mentoring	Identified minimum technical specification in terms of latency and frame rate

### Q3. How do these systems impact the acquisition of surgical skills, anatomical precision, and procedural competence?

The effectiveness of surgical telementoring systems can be evaluated based on the impact on surgical dexterity, tool-handling proficiency, and decision-making capabilities among the trainees

**P:** Trainee surgeons and medical students in LMICs

**I:** AI-assisted telesurgery, VR-based surgical simulation, and AR-enhanced telementoring

**C:** Traditional hands-on surgical skill acquisition

**O:** Increased surgical precision, reduced procedural errors, and faster skill acquisition

Table 6 summarizes the PICO evaluation for research Q3.

**Table 6:** PICO analysis to address research Q3

Ref	P	I	C	O
[21]	Surgical laparoscopic trainees	5G-enabled telementoring with real-time HD video	Traditional non-mentored training	Increased surgical precision, reduced errors
[37]	Surgeons and trainees in Tanzania	Structured video-based telementoring and mentorship	Pre-intervention period	Higher competency and procedure completion rates
[41]	Junior surgeons in LMICs	AR-based guidance with holographic overlays	No AR support	Improved tool handling and procedural accuracy
[42]	LMIC healthcare providers	Low-cost AR system for procedural skill training	Standard video training	Higher task success rate and improved confidence
[29]	LMIC surgical trainees	Remote telesimulation (GLAP platform)	Local simulation only	Improved laparoscopic skill and higher objective structured assessment of technical skills (OSATS) scores
[28]	Orthopedic trainees learning arthroscopy	AR-enhanced remote mentoring (SurgTime)	Face-to-face mentoring	Faster skill acquisition and better technical performance
[10]	Medical trainees (simulation-based)	Interactive VR training system	No VR exposure	Enhanced dexterity and improved psychomotor performance
[44]	Novice medical students	Remote suturing telementoring	Traditional in-person teaching	Enhanced suturing skill outcomes

### Q4. What outcomes are used to assess the effectiveness of telementoring in surgical training?

Qualitative and quantitative performance metrics are key in evaluating the effectiveness of surgical telementoring systems and programs.

**P:** Trainees in telementored surgical education programs

**I:** Performance evaluation metrics such as AI-driven analytics, procedural accuracy, and training duration

**C:** Conventional in-person surgical evaluation methods

**O:** Higher efficiency in surgical procedures, improved retention of skills, and greater trainee confidence, especially through complex procedures

Table 7 highlights the impact of quantitative and qualitative assessment tools and rating scales such as OSATS and Global Operative Assessment of Laparoscopic Skills (GOALS).

**Table 7:** PICO analysis to address research Q4

Ref	P	I	C	O
[21]	Surgical trainees	5G-enabled telementoring platform	Traditional or no mentoring	OSATS scores, error rates, latency benchmarks
[41]	Junior surgeons in LMICs	AR-based telementoring via HoloLens	No AR support	Tool tracking precision, task completion time, user ratings
[29]	Surgical trainees in LMICs	Online telesimulation (GLAP platform)	Local simulation	Performance scores, accuracy, and procedure success rate
[42]	LMIC healthcare workers	AR-based procedural teaching system	Video-only training	Task completion rate, error count, trainee usability score
[13]	Rural physicians	Real-time procedural telementoring	No telementoring support	Critical error rates, task success, and confidence scores
[28]	Orthopedic trainees	AR-enhanced telementoring	In-person mentoring	OSATS ratings and session feedback quality
[32]	Medical trainees	VR-based procedural simulation	No VR training	GOALS scores, tool path efficiency, psychomotor skills metrics
[44]	Medical students	Remote video-based suturing training	Classroom teaching	Improved task accuracy, error count, and satisfaction ratings
[37]	Surgical trainees in Tanzania	Onsite with remote video mentoring	Pre-intervention baseline	Case volume, complication rates, and confidence index
[44]	Novice learners	Remote mentoring in basic suturing	Traditional in-person instruction	Error count, time to task, and technique rating

### Q5. Where do current systems fall short, and how might modern communication technologies, e.g., 5G, edge computing, and immersive modalities, such as AR/VR and haptics, address these limitations?

Modern network technology, such as 5G, can enhance telementoring by reducing latency, improving video resolution, and enabling real-time haptic feedback. However, its deployment in LMIC regions is still lacking due to the high cost of equipment and an underdeveloped communication infrastructure.

**P:** Remote surgical trainees

**I:** 5G-powered ultra-low latency networks, AI-driven video analytics, and haptic-enabled telementoring systems

**C:** Existing telehealth infrastructures with lower bandwidth

**O:** Enhanced surgical guidance, seamless remote collaboration, and improved efficiency

Table 8 presents the PICO analysis focusing on the capabilities of modern communication technologies in advancing telementoring systems.

**Table 8:** PICO analysis to address research Q5

Ref	P	I	C	O
[21]	Surgical trainees	5G-enabled laparoscopic telementoring	Wi-Fi/4G telementoring	Latency $<100$ ms, improved image and video clarity, and real-time responsiveness
[45]	General healthcare users	Review of 5G deployment in surgical systems	4G/Wi-Fi communication systems	Higher resolution, faster transmission, and multi-user scalability
[13]	Rural emergency surgeons	Real-time telementoring highlighting bandwidth and delay issues	Traditional video conferencing	5G supports AR and haptics responsiveness
[1]	LMIC surgical teams	Projection of future 5G robotic telesurgery	Satellite and low-bandwidth systems	5G essential for latency-sensitive telesurgery applications
[41]	Junior surgeons	AR-based guidance tested on Wi-Fi vs 5G networks	Wi-Fi network baseline	Smoother frame rates, better AR synchronization under 5G
[42]	Healthcare trainees in LMICs	AR procedural training on limited bandwidth	No 5G, hence lower streaming quality	Suggested 5G would improve visual fidelity and reduce lag
[46]	Robotic surgery trainees	Real-time digital twin task execution platform	Local network or pre-5G infrastructure	5G needed for high-fidelity remote mirroring

## Results synthesis and discussion

The following key themes emerged in the evaluation of this systematic literature review, highlighting the evolving landscape of telementoring-based surgical training:

### Telementoring as a scalable model for remote surgical training

Effective and interactive telementoring systems leverage real-time telestration, AI-driven performance analytics, and reliable bidirectional voice and video communication to enhance surgical learning by trainees over a distance. The outcomes in the reviewed studies demonstrate significant enhancements in surgical precision, decision-making, and trainee confidence while addressing the scalability limitations of in-person mentorship. Some of the notable telementoring systems evaluated include the following:

- SurgTime Platform presented in Ref. [28] utilizes a PTZOptics 20X-NDI Broadcast Camera, an HD-DVI video feed, and a cloud-based interface enabling real-time telesurgical training. The system facilitates live annotation, with bidirectional voice communication, and 4K UHD surgical video transmission. Furthermore, it supports AI-enhanced intraoperative guidance. In the data stream transmissions, the system employs Real-Time Messaging Protocol (RTMP) and Web Real-Time Communication (WebRTC) protocols for low-latency and interactive live video broadcast streaming. The outcomes of this system include improved surgical precision, reduced complications, and enhanced mentor-mentee interaction. However, its widespread adoption, especially in underserved regions, is limited by high-bandwidth requirements, i.e., a minimum 100 Mbps stable network connection, low latency, i.e., $\sim$ 250 ms, and variability in user adaptability.
- GLAP Virtual Telesimulation Model presented in Ref. [29] utilizes biweekly virtual simulation-based laparoscopy training, incorporating real-time video conferencing, hands-on simulation exercises, and mentorship from remote experts. The system uses High-Efficiency Video Coding (HEVC).265 compression for bandwidth-efficient video streaming and AI-driven haptic feedback mechanisms, which enhance psychomotor skills acquisition. The HVEC.256 provides twice the compression efficiency of H.264 while maintaining the same video quality. It achieves this by using advanced motion compensation, improved intra-frame prediction, and larger coding block sizes, making it ideal for high-resolution (4K/8K) streaming in bandwidth-constrained environments. The outcomes of this system show significant skill improvement in laparoscopic procedures and increased participant confidence. However, deployment in rural hospitals is limited due to unstable internet connections. Additionally, the reliance on synchronous mentor availability hinders its scalability in under-resourced settings.
- Surgical mentorship models presented in Ref. [1] implemented structured mentorship networks integrating live surgical observation, virtual case discussions, and automated proficiency tracking via deep learning-based motion assessment. The deep learning module tracked trainee movements and performance on surgical tasks, and offered corrective suggestions and real-time feedback, and assessment. The outcomes of this system include increased adherence to surgical safety protocols and improved patient outcomes. However, this system requires substantial financial investment in AR/VR-compatible hardware and continuous mentor availability for long-term engagement, both of which may be limited in resource-constrained settings.

Table 9 provides a comparative analysis of the abovementioned telementoring systems, their technical specifications, performance metrics, and limitations.

**Table 9:** Comparison of modern telementoring systems: technical specifications and performance

System	Video	Latency	Bandwidth	AI features	XR support	Limitation
SurgTime	4K UHD, 60 fps	250 ms	100 Mbps+	AI-guided annotations	No	High bandwidth, latency
GLAP	1080p, 30 fps	180 ms	50 Mbps	AI-driven haptics	Yes	Internet instability
Surgical Mentorship Model	4K UHD, AR overlay	150 ms	75 Mbps	AI proficiency tracking	Yes	Costly, mentor dependency

### The role of 5G in enhancing surgical telementoring

5G network technology provides ultra-low latency ($<10$ ms), enhanced bandwidth (up to 1 Gbps per session), massive device connectivity, and high network reliability (99.9999%), making it ideal for real-time telesurgery applications. The ability to transmit high-definition video, real-time imaging, and haptic feedback with near-instantaneous response times significantly enhances the effectiveness of remote surgical training, as the trainer and trainee surgeon can interact as if they are in the same location. The following existing implementations illustrate the impact of 5G-enabled systems in advancing surgical mentorship.

- 5G-enabled telerobotic ultrasound in Ref. [47] implemented an MGIUS-R3 robotic ultrasound system with six degrees of freedom. It was controlled remotely via a 5G low-latency network. This system achieved high diagnostic accuracy and real-time image streaming. Despite the effectiveness of this system in telementoring, the initial setup costs as high, and dependency on stable 5G network coverage presents a challenge where network infrastructure is underdeveloped.
- Microsoft HoloLens AR Telementoring presented in Ref. [41] integrated Microsoft HoloLens mixed reality headset with a 3D tracking module, allowing remote mentors to overlay surgical images and guidance in real-time on the trainee’s view. This setup resulted in improved depth of perception, high accuracy in remote imaging, and improved instrument handling precision among the surgical trainees. However, the system lacked haptic feedback and field-of-view constraints within the AR interface, hindering full immersion and perception of the surgical field by the trainees.

To optimize 5G-powered telementoring, the following innovations can be integrated into the network architecture:

1. **Dedicated network slices for surgical applications:** In 5G, a network slice is a virtual, end-to-end partition built on a shared physical network infrastructure that is tailored to meet specific key performance metrics, security, or functional requirements for a particular service or application. Customized network slices can be allocated for telementoring, ensuring consistent low-latency, high-bandwidth communication. In addition, network slicing can achieve prioritization of latency-sensitive surgical training data streams, for improved synchronization between remote mentors and trainees.
2. **Fixed wireless access (FWA)** can be adopted to expand connectivity in areas where fiber deployment is a challenge. FWA is tailored home or business broadband delivered over a 5G mobile network instead of wired lines, such that a nearby 5G cell connects wirelessly to a customer premises device, such as a router, which then provides Wi-Fi/Ethernet. When deployed, this approach bridges the gap between urban surgical centers and remote hospitals, offering fiber-like speeds without the need for extensive infrastructure.
3. **Open-source based private 5G networks** for low-cost, controlled, high-performance communication, which enable on-premise private 5G networks within surgical facilities to ensure uninterrupted, secure, and tailored communication for telementoring sessions. Such networks can be optimized to support high-definition surgical training video, AR-based mentoring, and real-time haptic feedback, independent of commercial mobile network providers. Private 5G networks, powered by open-source platforms like OpenAirInterface (OAI), Software Radio System Radio Access Network (srsRAN), and Magma, allow healthcare institutions in LMICs to deploy localized, cost-effective 5G infrastructure without relying on commercial telecommunication operators, which can often be expensive and generic.
4. **Edge computing** for real-time data processing by deploying Multi-Access Edge Computing (MEC) nodes at strategic locations near surgical facilities will process real-time AI analytics, motion tracking, and haptic feedback locally. MEC is a computing paradigm where compute and storage nodes are placed very close to end users so that applications can run with ultra-low latency, high bandwidth, and local data processing. In surgical telementoring, this distributed approach prevents delays caused by routing data to centralized cloud servers, allowing instantaneous mentor-mentee interaction. It can also improve real-time AR/VR rendering, video analytics, and AI inference and assessments.
5. **Redundant connectivity** for reliability can be provided for critical telesurgery training procedures. Redundant links ensure that communication and data exchange are not disrupted when one link goes down. Hence, in rural and underserved regions where fiber infrastructure is underdeveloped or unavailable, the 5G network can be integrated with low-earth orbit satellite communication links, which are more reliable and readily available in rural regions. Such redundant links can ensure end-to-end availability and failover capabilities in case of terrestrial network disruptions.

### Extended reality in surgical telementoring

Extended reality (XR), comprising VR, AR, and MR, enables an interactive, high-fidelity, and immersive learning experience for surgical trainees. These technologies enhance spatial awareness, cognitive load management, and technical skill acquisition on virtual interfaces. However, the effectiveness of XR-based telementoring systems is highly dependent on network infrastructure, data transmission latency, and real-time rendering capabilities.

Some key implementations in this domain include:

- VR-enhanced training presented in Ref. [10] improved cognitive retention and technical accuracy in laparoscopy and robotic surgery training. VR-based simulations create a fully immersive digital environment, allowing users to practice surgical procedures in a simulated setting without real-world risks. VR systems often use haptic feedback, motion tracking, and AI-driven assessments to improve precision.
- AR-Based remote training outlined by Refs. [42] and [41] enabled real-time interactive mentorship, reducing learning curves for the trainees. AR overlays digital elements (holograms, annotations, and guidance) onto the real-world surgical field, improving depth of perception.
- In Ref. [12], an XR telementoring prototype where a remote mentor uses an optical see-through HMD to view the live 3D endoscopic scene and deliver stereo, occlusion-aware guidance overlays on the trainee’s robotic console feed is presented. The pipeline streams stereo endoscopy, reconstructs a 3D scene for the mentor, captures hand and virtual-tool inputs, and returns a time-synchronized overlay to the console. Quantitative tests on a controlled network reported accurate overlay registration and end-to-end latency on the order of a few hundred milliseconds ($\le$ 300 ms). A user study showed higher immersion, depth perception, and confidence versus conventional 2D video and telestration baselines.

Despite the benefits of integrating immersive technologies into telementoring systems, high-end VR/AR/MR headsets, haptic feedback devices, and computing systems remain expensive, limiting accessibility in LMICs. This calls for developing cost-effective, lightweight solutions tailored for resource-constrained environments. XR-based telementoring requires real-time data exchange, making network reliability a critical factor. Additionally, while haptic gloves and robotic-assisted surgery have improved tactile simulation, real-time force feedback in telesurgery remains a challenge.

### AI-driven surgical training and automated assessment

AI-powered machine learning models and deep neural networks enhance training by offering real-time skill assessment, automated feedback, and precision-guided surgical decision support between the mentees and the mentors. For example;

- AI-integrated Teleophthalmology presented in Ref. [48] showed an enhanced diagnostic accuracy via real-time AI-enabled image analysis, which is useful in helping the trainees to promptly identify patterns and features that may be useful in decision-making during a surgical procedure.
- AI-based performance analytics presented in Ref. [8] enabled automated tracking of skill progression in surgical simulations.

While AI-enabled systems in telementoring improve skill assessment and inference, computational requirements, validation concerns, and regulatory concerns in clinical decision-making need to be evaluated. In addition, AI models must be trained on diverse, high-quality datasets to ensure generalizability across different surgical procedures and patient demographics. A major challenge is the limited availability of annotated surgical datasets in LMICs, necessitating federated learning techniques for collaborative AI model training across institutions [49]. Several AI models and algorithms are particularly suitable for surgical telementoring for processes such as real-time assessment, feedback, and predictive analytics. Such models include:

1. Deep learning-based skill assessment, which includes convolutional neural networks (CNNs), would be ideal for gesture recognition and surgical tool tracking in laparoscopic and robotic surgery training. Furthermore, this integration will facilitate automated skill evaluation in telementoring [8].
2. Reinforcement learning for robotic assistance, including deep reinforcement learning (DRL), allows AI systems to adapt to new surgical tasks and provide personalized feedback. For instance, in Ref. [50], DRL is proposed for adoption in telesurgery practices to guide the adjustment of grip force and precision movements during surgical mentorship.
3. Recurrent Neural Networks (RNNs) can be used to assist in predicting the next surgical step and detecting errors in real time. This is useful because AI can monitor intraoperative movements and suggest corrections to the mentee, therefore reducing procedural errors in remote telementoring setups [30].
4. AI-powered speech and tactile feedback systems can adopt natural language processing (NLP) algorithms that enable voice-guided AI mentorship during surgery. Furthermore, AI-enhanced force-feedback systems can enhance the prediction and refinement of tactile precision in robotic telesurgery training [51].

### Digital simulation and telesurgery for procedural training

Simulation models allow hands-on surgical practice without patient risk. The combination of low-cost AI-driven adaptive learning and cloud-based telesurgery offers scalable training solutions that would be ideal for LMICs. Examples of implemented simulation systems include:

- SIMISGEST-VR system presented in Ref. [52] showcases a low-cost VR-based simulator for minimally invasive surgery training. This system enhances laparoscopic skill acquisition using motion tracking, force feedback, and AI-guided assessment.
- Hybrid physical simulation models in Ref. [25] entail 5 to 20 models for pericardiocentesis and thoracostomy training, utilizing 3D-printed anatomical structures combined with digital haptic overlays to simulate procedural realism.

### Economic and sustainability considerations

Cost-effectiveness in surgical telementoring should consider both the total cost of ownership, such as equipment, network access, and computing, and operating costs, such as training, bandwidth, maintenance, and system downtime. Reviews of existing telementoring in surgical care repeatedly identify connectivity fees and platform costs as recurrent expenditures for hospitals with constrained budgets and competing clinical priorities [11, 13]. While not all telementoring requires robotics, evidence from robotic programs in resource-constrained settings highlights high capital, maintenance, consumables, and training costs as persistent barriers. This situation also applies to advanced imaging, XR, and specialized peripherals often bundled with telementoring frameworks [6, 33].

Sustainability of telementoring systems may be enhanced through hybrid funding models that combine governmental support, non-profit contributions, and local user fees, ensuring long-term viability beyond initial grants. Many LMICs operate with unreliable or absent grid electricity, forcing programs to budget for power conditioning, battery, and increasingly solar hybrid microgrids to ensure uptime for live telementoring sessions [9, 53]. On the network side, studies document unstable bandwidth, latency spikes, and video degradation, which drive hidden operational expenses due to over-provisioning, bonded links, or redundant internet service providers, and can limit educational benefits if not mitigated [11, 13]. In the COVID-19 era, telemedicine scale-ups further showed that short-term donor or emergency funds can boost adoption, but long-term continuity stalls without embedded financing and institutional policies [54].

Hence, a sustainable telementoring system model would entail the following aspects [11, 13, 21]:

- Fit-for-purpose design including smartphone-centric capture, adaptive bitrate for low throughput scenarios, edge processing to cut backbone requirements.
- Modular Procurement whereby adoption starts with core telepresence and telestration, then XR and haptics can be added as capacity grows.
- Pooled and subsidized bandwidth and negotiated service level agreements with network operators at regional and national levels to lower running costs.
- Local capacity building of biomedical and information technology (IT) support for maintenance and operation, alongside a train-the-trainer program that will ultimately reduce downtime and external contractor costs.

In addition, training program governance should align with national digital health strategies, i.e., privacy, security, interoperability, and data residency, to avoid regulatory pitfalls that can disrupt cross-border mentorship or inflate compliance costs [26, 55, 56]. To accurately evaluate the cost, sustainability, and economic justification for telementoring systems, prospective and iterative evaluations should be done. This may entail pairing pilots with cost-effectiveness analyses, such as mentor time saved, patient transfer avoidance, shorter learning curves, complications reduced, and an increase in the number of surgical and medical experts in LMICs. Further evaluation can include reports on lifespan and utilization of devices to inform depreciation and refresh cycles [11, 33].

### Ethical and regulatory considerations

Ethical deployment of telementoring systems in LMICs ensures that training standards, patient safety, privacy, accountability, and equity are followed while navigating cross-border law and health-data governance. Given that clinical advice traverses jurisdictions, telementoring programs can assume that the patient’s or trainee’s location determines the applicable law and licensing; hence, remote mentors typically need explicit authorization or institutional agreements that lawfully cover supervision and education in that jurisdiction [26, 56]. Many LMIC programs must also navigate regional data-protection baselines, for instance, in Africa, the African Union Malabo Convention on Cyber Security and Personal Data Protection entered into force in June 2023, establishing common principles that national laws increasingly reflect [57]. Reviews of surgical telemedicine in LMICs also emphasize the need to define roles and liability up front, i.e., who is the responsible local clinician of record, what constitutes advice versus direction, and how escalation is handled so that mentorship conforms to clinical accountability at the point of care [11, 13].

Privacy, security, and data governance are critical such that high-fidelity video, audio, and telestration and overlays qualify as personally identifiable health data and must be protected under national laws e.g., the United States of America Health Insurance Portability and Accountability Act (HIPAA)-aligned rules in some countries and, for cross-border flows, frameworks such as the European Union’s General Data Protection Regulation (GDPR) principles that include data minimization, purpose limitation, storage limitation, integrity and confidentiality, and appropriate transfer mechanisms [26, 55]. Telementoring systems should also implement end-to-end encryption, strict access control and role-based permissions, audit trails, consent for live streaming and recording, and clear retention and de-identification policies. LMIC scoping reviews report that fragmented policies often leave hospitals vulnerable to ad hoc platform choices and insecure workarounds, highlighting the need for institutional policies and national guidance [11]. Where data residency or localization rules exist, platforms must support in-country storage or contracted processing that complies with local law [26, 55].

In addition, equity and fairness considerations should be integrated throughout all stages, including system design, consent, language access, and the allocation of scarce mentoring time [26, 55, 56]. Evidence from LMIC telemedicine implementations during COVID-19 shows that programs risk increasing disparities when bandwidth, devices, or digital literacy are unevenly distributed. Ethically, implementations should include access provisions, e.g., on-site hubs, shared equipment, training, and monitoring for differential benefit across sites and populations [11, 54]. Where AI or analytics are embedded, e.g., skill scoring, and guidance prompts, governance should incorporate human oversight, transparency of model limits, and monitoring for bias, as outlined in WHO digital health guidance [55].

## Towards an ideal telementoring system for surgical training in LMICs

In this work, we did not conduct an empirical validation or pilot deployment. As a systematic review, our findings rely on published evidence, which limits direct inference about local feasibility, workflow integration, and cost sustainability in LMIC sites. To address this, we specify a prospective pilot design below to test the framework under operational constraints.

**Fig. 3:**
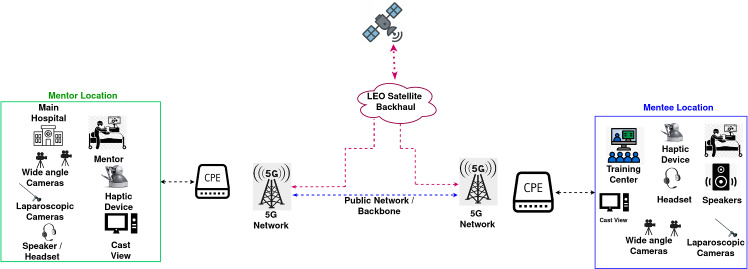
Overview of a 5G-enabled telementoring system

Figure 3 illustrates a framework for developing a telementoring system for improving surgical skills capacity in LMICs. As shown, an ideal telementoring system includes both mentor-side and mentee-side components, which are interconnected by a communication network infrastructure.

### Key components

#### Mentor location

This is the site where the mentor surgeon performs procedures in real time while guiding remote mentees. They need the ability to demonstrate surgical procedures and techniques via live video or recorded sessions. Typical components at this site include:

- Image and video capturing equipment: The mentor site is equipped with general and laparoscopic cameras, and audio systems to capture high-definition video streaming details of surgical procedures in real time. The audio system should allow two-way audio for real-time discussions between mentors and mentees. Remote pan-tilt-zoom (PTZ) cameras may also be incorporated for mentor control. PTZ cameras allow remote mentors to adjust viewing angles, zoom in on surgical sites, and track instrument movements dynamically. This capability is essential for procedural clarity, ensuring that critical anatomical structures and surgical maneuvers are visible.
- Haptic devices are used by the mentor surgeon to provide real-time force feedback, enhancing remote tactile sensing for precise tool handling. These devices allow mentor surgeons to “feel” interactions as they guide mentees and for them to be fully immersed in the mentee’s real environment through touch sensations.
- The cast view enables real-time screen sharing and visualization of images, procedures, or AR overlays, enabling the mentor to input annotations on the video stream they receive from the mentee’s location.
- AR/VR interfaces to enable digital overlay annotations, holographic guidance, and telestration to enhance surgical comprehension and decision-making.

#### Network domain

Network communication infrastructure should support reliable and real-time interaction between the mentees and mentors. Ideally, the network system should be comprised of;

- 5G private access network integrated with public backbone and satellite. A private 5G network can be deployed within the hospital or training facility for low-latency, high-bandwidth local communication. The availability of open-source 5G software frameworks, such as OpenAirInterface 5G, srsRAN, Open5gs, and commercial-off-the-shelf radio equipment, has made it easy to achieve a low-cost, optimized, and flexible network. To interconnect the mentee and the mentors who are in geographically dispersed locations and beyond reach of fiber backbones or even consistent broadband coverage, Low Earth Orbit (LEO) satellite backhaul, such as SpaceX Starlink, OneWeb, Telesat LEO, and Amazon’s Kuiper, provides an ideal solution, where public Internet and fiber infrastructure may be lacking or underdeveloped. LEO satellites orbiting at $\sim$500–1200 km altitude reduce latency compared to traditional geostationary satellites and can provide broadband Internet with round-trip latency on the order of 30–50 ms, making them suitable for interactive applications like video conferencing and potentially even surgical telementoring [58, 59]. In this case, the mentor and the mentee sites can each have 5G private networks, and the backhaul can be realized using a LEO satellite. Modern LEO constellations aim to offer 100–200 Mbps or more per user terminal. Even in sparsely populated regions, a single user dish (with a clear sky view) can get comparable speeds to suburban fiber or cable, with tens of Mbps sustained. Given that LEO satellites move quickly across the sky, each link typically hands off from one satellite to the next, allowing consistent throughput. This aspect is beneficial where building fiber to every remote location is cost-prohibitive. Over time, if fiber or microwave links become available in target remote regions, the satellite terminal can be repurposed for backup or redundant service.
- Network slicing capabilities for traffic differentiation and prioritization need to be implemented. Network slicing allows the creation of multiple logical channels on a shared physical infrastructure, where each logical channel can meet the QoS and serve a specific use case. Given that the network may be shared with other users, it is important to slice the network so that the Telementoring application has an isolated slice with QoS guarantees for the audio, video, and haptic data traffic. Through network slicing, policies implementing traffic prioritization can be enforced to ensure that the telementoring session is undisturbed [60]. Furthermore, given the diverse nature of traffic expected in a telementoring use case, a multi-layered network slicing approach can be implemented, as follows:
  - A primary slice dedicated to general surgical training data transmission, ensuring ultra-reliable low-latency, high availability, and high throughput communication.
  - Sub-slices for specific data may be configured to handle the different traffic whose performance requirements are distinct. For instance, a haptic data sub-slice to handle traffic that requires sub-1 ms latency for real-time force feedback synchronization. On the other hand, a high-definition sub-slice can be configured for traffic that demands high bandwidth, such as HD video streams, to ensure low-jitter transmission and seamless 4K/8K surgical training video feeds. In addition, an AI-powered analytics sub-slice that allocates resources for real-time machine learning-driven skill assessments and procedural evaluation can be implemented.
- Edge computing capability for latency optimization, which entails deploying multi-access edge computing nodes at strategic locations to allow AI-based analytics and pre-processing of latency-sensitive data, such as haptic data streams near the source, reducing end-to-end latency.

Ultimately, the performance of the network domain should be validated by measuring the KPIs during a telementoring session and comparing them with the metrics outlined in Table 10.

**Table 10:** Technical requirements for an interactive telementoring system

Requirement	Value	Justification	Ref
End-to-end latency	$<10$ ms	Guarantees real-time telesurgery and haptic feedback	[21]
Video Resolution	4K UHD at 60 fps	Ensures high-definition visualization	[28]
Frame Rate	$\ge 60$ fps	Reduces motion blur and improves detail clarity	[10]
Audio Quality	48 kHz, 16-bit	Enables clear, low-latency communication	[42]
Haptic Feedback Latency	$<1$ ms	Required for precise tactile feedback in robotic telesurgery	[41]
Network Bandwidth	Upto 1 Gbps	Supports multiple video, audio, and haptic data streams	[1]
Packet Loss	$<0.001$%	Ensures stable and error-free transmission	[8]
Jitter	$<30$ ms	Maintains smooth video and audio transmission	[41]
Encryption	AES-256	Protects training data and ensures cybersecurity compliance	[5]

#### Mentee location

Mentee location essentially comprises interactive learning tools for multi-user training sessions for remote participants.

- Bidirectional audio system to ensure clear audio communication between mentors and mentees.
- Haptic devices allow trainees to experience real-time touch-based feedback. Equipping the training with haptic devices enables the trainees to feel and replicate the consistency and pressure against tissues and organs being manipulated by the training surgeon, and as a result, enables them to develop and advance their psychomotor skills.
- Cast view and screen sharing for displaying live procedures, 3D models, and annotated surgical views.
- AI-based surgical analytics provides automated skill assessment, error detection, and procedural guidance for trainees based on machine learning-driven real-time feedback.
- Image and video capturing equipment, including general and laparoscopic cameras to capture high-definition video streaming details of surgical procedures in real time.

## Future research directions

While existing systems have created opportunities to advance surgical skills globally, the effectiveness of these training models would benefit from achieving a fully interactive and immersive experience, as if it were in-person training. This review synthesizes published works without conducting new empirical trials. As such, conclusions are contingent on the quality and scope of existing studies, which may be subject to publication bias. Therefore, future work should involve controlled pilot deployments to evaluate latency, haptic feedback quality, and surgical performance outcomes, ideally in collaboration with hospitals in both high- and low-resource settings.

Other than developing robust and cost-effective networking and technology solutions, the following aspects need further research:

- Current evaluations are predominantly short-term and focus on immediate procedural accuracy or confidence improvements. Future research should investigate long-term surgical competency retention and patient outcomes over time to fully validate the clinical efficacy of telementoring systems in LMICs.
- The absence of standardized metrics and universal training frameworks limits cross-study comparisons and reproducibility. Future work should aim to develop and validate quantitative and qualitative assessment instruments and metrics, such as global benchmarks for technical skill proficiency, mentoring effectiveness, and trainee progression, tailored for remote surgical education.
- Telementoring deployments should also focus on cost management so that the systems are adapted to LMIC-specific infrastructural and financial constraints. These may include interventions such as modular AR and VR kits, smartphone-based platforms, low-cost renewable power solutions, and open-source network architectures that reduce total cost while maintaining functional performance.
- Satellite-enabled and 5G-extended infrastructure in areas lacking terrestrial internet infrastructure is essential. Studies should explore the impact of network slicing, latency optimization, and data prioritization techniques for maintaining QoS during a live surgical mentoring session.

## Conclusion

This systematic literature review highlights the transformative potential of surgical telementoring in addressing surgical training challenges in LMICs. Through a structured PICO-based analysis, we identified existing platforms such as SurgTime, Project ECHO, and 5G-assisted telesurgery as effective in enhancing skill acquisition, knowledge retention, and access to expert mentorship in resource-constrained settings.

Emerging technologies, particularly 5G, AI, and XR, offer promising capabilities for improving latency, bandwidth, and interactivity in remote surgical guidance. However, critical barriers remain, including infrastructural limitations, regulatory uncertainty, high implementation costs, and cybersecurity risks. To address these challenges, future telementoring systems should integrate open-source solutions, cost-effective 5G and satellite communication frameworks, AI-assisted evaluations, and edge computing to enable low-latency, secure, and scalable deployments. More research should be done to ensure that the ethical and legal frameworks that govern cross-border mentorship, data privacy, and system accountability are followed. Advancing technical and functional capabilities will pave the way for resilient, accessible, and globally deployable telementoring systems that can improve surgical capacity, education, and patient outcomes in underserved regions. While existing surgical telementoring systems in LMICs demonstrate positive outcomes in the reviewed case studies, pilot testing is required to validate the proposed framework under real-world operational conditions. In addition, the lack of large-scale, randomized controlled trials limits the generalizability of current findings.

## Data Availability

No datasets were generated or analyzed during the current study.
